# Ups and Downs: Mechanisms of Repeat Instability in the Fragile X-Related Disorders

**DOI:** 10.3390/genes7090070

**Published:** 2016-09-21

**Authors:** Xiao-Nan Zhao, Karen Usdin

**Affiliations:** Section on Gene Structure and Disease, Laboratory of Cell and Molecular Biology, National Institute of Diabetes, Digestive and Kidney Diseases, Building 8, Room 2A19, National Institutes of Health, 8 Center Drive MSC 0830, Bethesda, MD 20892-0830, USA; xiaonan.zhao@nih.gov

**Keywords:** Fragile X syndrome, Fragile X-related disorders, repeat expansion, base excision repair, mismatch repair, transcription coupled repair

## Abstract

The Fragile X-related disorders (FXDs) are a group of clinical conditions resulting from the expansion of a CGG/CCG-repeat tract in exon 1 of the *Fragile X mental retardation 1 (FMR1)* gene. While expansions of the repeat tract predominate, contractions are also seen with the net result being that individuals can show extensive repeat length heterogeneity in different tissues. The mechanisms responsible for expansion and contraction are still not well understood. This review will discuss what is known about these processes and current evidence that supports a model in which expansion arises from the interaction of components of the base excision repair, mismatch repair and transcription coupled repair pathways.

## 1. Introduction

This year marks 25 years since the genetic basis of Fragile X syndrome (FXS) was shown to be the increase in size or expansion of a CGG/CCG-repeat tract in the 5′ untranslated region of the *Fragile X mental retardation 1* (*FMR1*) gene [1]. This expansion occurs from a predisposed premutation (PM) allele with 55–200 repeats to a full mutation (FM) allele that has at least 200 repeats and may in fact have gained hundreds if not thousands of additional repeats. At the time this sort of mutation was unusual, shared only with another disorder X-linked spinal and bulbar muscular atrophy (SBMA; also known as Kennedy’s disease) whose genetic basis, expansion of a CAG/CTG-repeat tract in the androgen receptor gene, was discovered the same year [2]. Today, we know that expansion mutations are the cause of more than 30 different human genetic disorders including myotonic dystrophy type 1 (DM1), Huntington disease (HD) and Machado-Joseph disease/Spinocerebellar ataxia type 3 (MJD/SCA3) that, like SBMA, involve CAG/CTG repeats, as well as Friedreich ataxia (FRDA) that involves GAA/TTC repeats (reviewed in [3]). However, many questions about these sorts of mutations remain unanswered. In this review we will try to address some of these questions. We will focus primarily on what we have learnt about repeat instability in FXS and the other FX-related disorders (FXDs), Fragile X-associated tremor/ataxia syndrome (FXTAS) and Fragile X-associated primary ovarian insufficiency (FXPOI) from studies of affected families and from Fragile X (FX) PM mouse models with a targeted insertion of 98–130 CGG/CCG-repeats [4,5]. However, where necessary we will also draw on the much larger body of work done in the other Repeat Expansion Diseases to help develop a more complete picture of these events.

Alleles with large numbers of CGG repeats are unstable, undergoing both expansion and contraction. However, there is an expansion bias with expansions outnumbering contractions by ~10:1 in both humans and in FX PM mouse models [4,5,6,7]. This is very different from the genome-wide microsatellite instability (MSI) seen in certain kinds of cancer. Both expansion and contraction are likely to contribute to the mosaicism seen in many carriers of *FMR1* alleles with large repeat numbers.

## 2. When and Where Does Repeat Instability Occur?

When and where expansion occurs is still somewhat controversial, but it is relevant for our understanding of the expansion process, since expansion in non-dividing cells like oocytes or neurons would suggest a mechanism that involves aberrant DNA repair or recombination rather than a problem with chromosomal replication, while expansion in dividing cells would be compatible with all three possibilities.

While large expansions are maternally transmitted in humans, males with FXS have only PM alleles in their sperm [8]. The presence of PM alleles in the sperm of FM males could be consistent with the idea that expansion occurs in the somatic cells of the embryo after differentiation of the primordial germ cells from the other cell lineages. It is also possible that expansion occurs earlier than that with selection against large expansions in sperm. Since a number of embryonic stem cells (ESCs) have been isolated in which expansion into the FM range has already occurred [9,10,11,12], expansion likely occurs prezygotically or in the very early embryo. This hypothesis is strengthened by the observation that no progressive increase in repeat number is seen in stem cells from PM carriers or in stem cells from carriers of unmethylated full mutations [13]. Expansion in the oocyte would be consistent with the maternal age effect that is seen for the risk of having a child with FXS [14]. It would also be consistent with the observation from a FX PM mouse model in which expansion risk is related to maternal genotype and not the genotype of the offspring [7]. Selection against large alleles in sperm would be consistent with the known difficulty in replication through large CGG/CCG-repeat tracts [15,16], a phenomenon that may be particular problematic during the rapid cell division that occurs during spermatogenesis.

**Figure 1:**
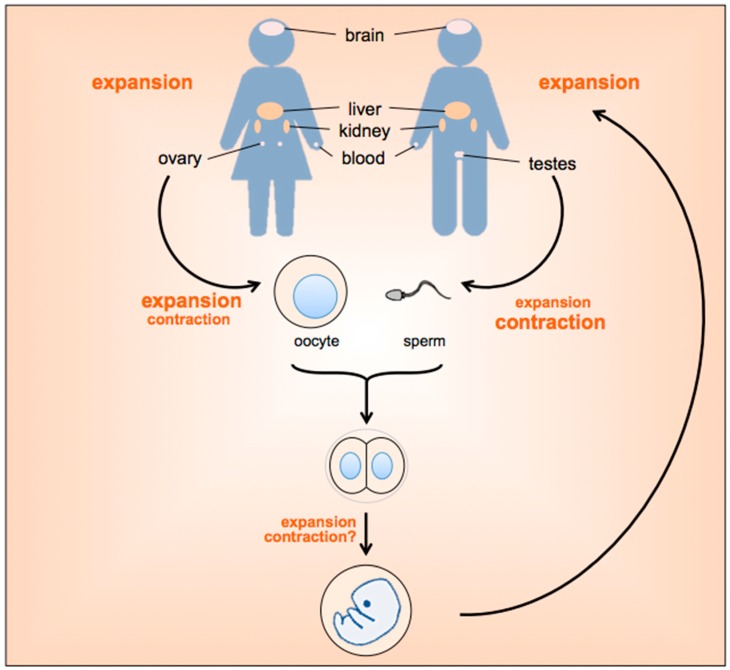
Timing and tissue distribution of expansions in the Fragile X-related disorders (FXDs). The data for this figure reflects a synthesis of data from humans and a mouse model of the Fragile X premutation (FX PM). Intergenerational expansions likely occur prezygotically and perhaps in the very early embryo. Both maternal and paternal expansions are observed. However, as the repeat number increases so the frequency of paternally transmitted contractions increases [19]. In contrast, expansions that generate larger alleles predominate in females, with the likelihood that a PM allele will be transformed into a full mutation (FM) allele reaching 100% as the repeat number approaches 90 [19]. In humans, somatic expansions have been observed in blood and brain. Work in the FX PM mouse suggests that expansions can also occur in organs like liver and kidney, with more somatic expansions being observed in males than females. No contractions have been seen in the somatic tissue of adult animals. The extent of expansion and contraction is indicated by the font size of the associated text.

However, as illustrated in Figure 1, some expansions may also occur in the early embryo as data from the mouse model suggests [7]. Furthermore, expansion has been observed in blood and brains of human PM carriers and in organs like liver, skin and kidney in the mouse model [17] although the biological consequences of these expansions is unclear. Expansions are also seen in terminally differentiated neurons in a mouse model of HD [18].

## 3. What Factors Affect Expansion Risk?

Expansion risk is directly related to repeat number. The smallest human allele known to have expanded into the FM range had 56 repeats, while 94% of alleles with >90 repeats expand into the FM range [19]. However, humans often have one or more AGG-interruptions at the 5′ end of the repeat tract that have a periodicity of 9–11 repeats. These interruptions reduce expansion risk [20], with the number of uninterrupted repeats at the 3′ end of the repeat tract being the best predictor of expansion risk [6]. However, even after accounting for the number of AGG-interruptions, carriers of alleles with 70–79 repeats with a family history of FXS underwent expansion to a FM 54% of the time, compared to 11% for carriers of similar sized alleles with no family history. For alleles having 80–89 repeats, the expansion risk increased to 88% for those with a family history while those without a family history underwent expansion only 33% of time [19]. Thus, it is likely that there are additional genetic factors that modify expansion risk.

As discussed below, a number of DNA repair factors that promote or protect against expansion have been identified in mice. By analogy with what has been found in a mouse model of HD [21,22], mutations that affect the efficacy of these proteins may affect expansion risk for some individuals. Indeed, single nucleotide polymorphisms (SNPs) in some of these DNA repair factors have been found to be associated with increased risk of expansion in DM1, HD and MJD/SCA3 [23,24,25].

Work in FX PM mice has shown that exposure to oxidizing agents that damage DNA also increases expansion risk [26], raising the possibility that oxidative stress may increase expansion risk in humans as well. We have also shown that female mice have a significantly lower risk of expansion than male mice [27]. Some of this reduced risk is related to the fact that expansion does not occur on the inactive X chromosome in our mouse model [27]. A retrospective analysis of human data suggests that the same is true in women with the PM [28]. Thus, transcription or the presence of the PM allele in a region of transcriptionally competent chromatin is required for expansion. However, other factors likely also contribute to the differences in expansion risk in male and females. These factors are not related to the effects of either testosterone or ovarian hormones or to differences in the levels of expression of the proteins thus far implicated in repeat expansion [27]. However, differential expression of a number of genes involved in the response to oxidative stress is seen between male and female mice with the PM. Given the contribution of oxidative stress to expansion, it is possible that this differential expression contributes to the higher expansion frequency in males [27].

## 4. What Is It about the Repeat that Makes It Unstable?

**Figure 2:**
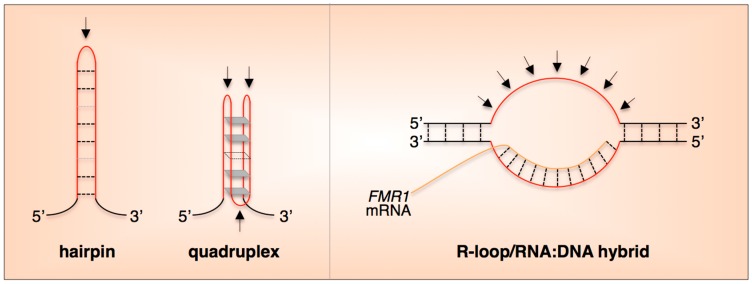
Generic representation of some of the unusual DNA structures described for the FX repeats. The arrows indicate single-stranded regions. Both DNA strands can form hairpins containing a mixture of Watson-Crick G•C base pairs (black dashed lines) and either G•G or C•C mispairs in the case of the CGG-strand and CCG-strand, respectively (gray dashed lines) [30,31,32,33,39]. Hairpin formation on one strand may facilitate hairpin formation on the other. This could result in the formation of Slipped DNA (S-DNA) [40] if the hairpins are offset. Quadruplexes may involve G4-tetrads and/or GCGC-tetrads (gray parallelograms) with C•C mismatches (dashed lines) [29,31,41,42,43]. The repeat tract can also form an R-loop containing an RNA:DNA hybrid formed between CGG-repeats in the transcript and the CCG-repeats in the DNA template [35,36,44]. In principle this hybrid could form in cis (perhaps co-transcriptionally) or in trans.

The FX repeats, like the repeats responsible for the other Repeat Expansion Diseases, form unusual DNA structures like hairpins, quadruplexes, Z-DNA and persistent R-loops as illustrated in Figure 2 [15,29,30,31,32,33,34,35,36,37]. The intrastrand structures could form any time the two strands of the repeat were unpaired, during replication or repair for example. R-loop formation may also provide an opportunity for intrastrand structures to form on the non-template strand. Resolution of the RNA:DNA hybrid in the R-loop could then allow the template strand to form secondary structures as well. As illustrated in Figure 2, the regions of single-strandedness present in most of these structures could predispose the repeat region to damage by a variety of DNA damaging agents. For example, guanines in the single-stranded loops of hairpins formed by CAG-repeats are prone to oxidative damage [38], and it is likely that guanines in the loops of hairpins formed by the FX repeats will be too.

Some of these structures also block DNA synthesis both in vitro and in vivo [15,16,31]. This has the potential to cause replication fork stalling and collapse. Furthermore, because of their repetitive nature and their propensity to form folded structures, the repeats are likely to be prone to strand-slippage during DNA synthesis. A 5′ slippage of the nascent strand with repriming from the slipped position could result in the incorporation of these supernumerary bases into the nascent strand, whilst slippage of the template strand with repriming further 5′ on the template could result in the nascent strand having fewer repeats than the template strand. These structures could also form on any strand-displacement products arising during replication or repair. Such structures are not processed properly by enzymes like the flap endonuclease FEN1 [45], and thus may result in these bases being incorporated into the nascent strand.

Since the CGG-strand of the FX repeat forms folded structures that are more stable than those formed by the CCG-strand [46,47], any effect of the repeat on DNA synthesis is likely to be dependent on which strand of the repeat is being synthesized. Since AGG interruptions would decrease the stability of secondary structures formed by the repeat, and limit where repriming could occur after strand-slippage had taken place, this could account for the ability of AGG interruptions to reduce expansion risk.

## 5. What Are the Current Models for Expansion?

A large body of work in bacteria and yeast has shown that the frequency of expansions and contractions can be affected by the orientation of the repeat relative to an origin of replication (ORI) or the distance of the repeat from this ORI [54]. This has led to the development of number of different models that invoke a problem with chromosomal replication as the source of expansions. For example, in ESCs from two FM carriers, replication through the repeat occurs from only a single ORI located telomeric to the repeat whilst in normal ESCs or in differentiated cells generated from both normal and FM ESCs, replication through the repeat uses ORIs located on either side of the repeat [11]. The single ORI active in FM ESCs would result in replication through the repeat such that the CGG-rich strand that forms the more stable secondary structures would be on the lagging strand nascent strand. This situation may be more likely to result in expansions. In contrast, in cells where both ORIs are used, it is suggested that expansions and contractions occur with equal frequency thus resulting in no net gain of repeats. Both of the FM cell lines used in this study carried an X chromosome belonging to the D haplotype group, a haplotype associated with a high risk of expansion [55]. Furthermore, they also carried a SNP that co-segregates with the subset of the D group with the highest expansion risk [55]. This SNP was located in the more centromeric ORI and it was suggested that this SNP accounted for the failure of this ORI to function in ESCs [56]. Whether a similar mechanism can account for expansion on chromosomes with different haplotypes is unclear.

However, since the available data suggests that expansions occur in cells like oocytes and neurons that do not divide, such a mechanism may not be responsible for expansion in some of the most disease-relevant cell types. Of course, there are other cellular processes that involve DNA synthesis that could result in expansion including DNA damage repair and recombination. While mutations in genes that affect repair of double-strand breaks have not been tested in the Fragile X-related disorders (FXDs), mutations in the catalytic subunit of the DNA-dependent protein kinase (DNA-PK), that is involved in non-homologous end-joining, did not affect expansions in a mouse model of DM1 [57]. Neither did mutations in the homologous recombination proteins RAD52 and RAD54 [57]. However, since the loss of RAD52 and RAD54 has only a minor effect on recombination in vertebrates [58,59,60], this finding does not definitively exclude recombination-based models.

**Figure 3:**
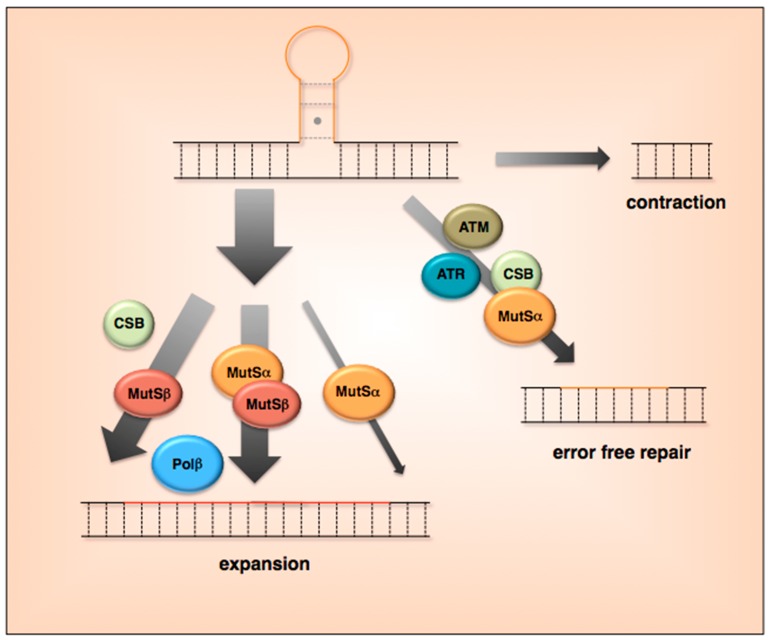
Types of repeat instability at the *Fragile X Mental Retardation 1 (FMR1)* locus. Evidence from a mouse FX PM model suggests that processing of the FX repeats during replication and repair can lead to either expansion, contraction or error-free repair. Expansion is by far the dominant mechanism with >80% of alleles showing expansion in male mice at 6 months of age [7]. About 2% of expansions involve the mismatch repair protein MutSα, with the remaining expansions involving either the mismatch repair protein MutSβ or a combination of MutSα and MutSβ [48,49]. DNA Polymerase β (Polβ), a polymerase essential for base excision repair, is also an important contributor to expansion with the Cockayne Syndrome Group B (CSB) protein, that is normally involved in Transcription Coupled Repair (TCR), playing an auxiliary role in older animals [50]. Contractions and error-free repair occur at about the same frequency, with error-free repair involving CSB [51], MutSα [49], as well as the DNA damage checkpoint proteins Ataxia Telangiectasia Mutated (ATM) and Ataxia Telangiectasia and Rad3-Related (ATR) [52,53]. Proteins involved in generating contractions have not yet been identified.

However, since as illustrated in Figure 3, proteins in various DNA repair pathways have been shown to be essential for expansion in mouse models of different Repeat Expansion Diseases, attention has focused in recent years on these other pathways. A number of proteins that are typically associated with mismatch repair (MMR), the repair of single base mismatches or small insertions/deletions (INDELs), have been shown to be essential for expansion. These include the DNA mismatch repair proteins MSH2 and MSH3, the constituents of the heterodimer MutSβ that recognizes small INDELs [7,48]. These proteins have also been implicated in mouse models for the CTG/CAG-repeat expansion diseases DM1 and HD [22,57,61,62,63,64]. MutSβ binds to the secondary structures formed by both sets of repeats and alters the properties of mismatch recognition [48,49,65]. This has led to the idea that MutSβ may be acting by promoting the formation and/or preventing the removal of hairpins generated during repair synthesis. We have also shown that the second MutS complex found in mammalian cells, MutSα, also contributes to expansion. However, since MutSβ is required for 98% of expansions, MutSα may contribute to expansion by facilitating the activity of MutSβ [49]. The fact that MutSα promotes MutSβ binding to the repeats may be one way in which MutSα contributes to the expansion process. A SNP in the *MSH3* gene is associated with increased expansion risk in DM1 [23] and a genome wide association study (GWAS) has demonstrated an association between age of onset of HD and the DNA mismatch repair protein MLH1, a protein with which MutSα and MutSβ interact [25]. Furthermore, knockdown of some of these MMR proteins in human cell models of different Repeat Expansion Diseases also reduces expansions [66,67,68]. Thus MMR proteins are likely to be important for expansions in humans as well. These data suggest that the very same proteins that protect the genome against MSI, are actually responsible for repeat expansion.

In addition, work in our lab has also demonstrated that Polβ, a DNA polymerase that is not involved in MMR but that is required for Base Excision Repair (BER), is also important, perhaps essential for expansion in a FX PM mouse model [69]. A role for BER is consistent with our observation that oxidative damage increases expansion risk [26], since BER is the major pathway by which oxidative damage to DNA is repaired. It is also consistent with the demonstration in the HD mouse model that DNA glycosylases responsible for early steps in the BER pathway also contribute to expansions [70,71].

We have also shown that Cockayne Syndrome B (CSB), a protein essential for Transcription Coupled Repair (TCR), plays a role in promoting expansions in mice [50,51]. In MJD/SCA3, a SNP in the *Excision Repair Cross-Complementation Group 6* (*ERCC6*) gene that encodes CSB has been found associated with increased risk of expansion, suggesting that CSB may be important in humans as well [24]. However, the loss of CSB only reduces the somatic expansion frequency in a limited number of organs in mice and only reduces the intergenerational expansion frequency in older mothers. Thus CSB is not essential for expansion. Since CSB is essential for TCR this suggests that TCR per se is not involved in the generation of expansions. Rather, CSB may be acting to promote expansions via its ability to participate in other DNA repair pathways perhaps when the factors essential for expansion become rate limiting.

Thus, the available data suggests that the components of three quite different DNA repair processes interact in some way to generate expansions. How this happens is not clear. One possibility is that transcription creates the opportunity for secondary structures to form on the non-template strand and that these secondary structures are prone to oxidative damage. The BER machinery then initiates repair, with Polβ facilitating strand slippage and strand displacement. This creates the opportunity for additional secondary structures to form. These structures are recognized by the MMR machinery that then processes them in an error-prone way to generate expansions. CSB may contribute to expansion via its ability to promote the activity of BER enzymes like 7,8-dihydro-8-oxoguanine-DNA glycosylase (OGG1), Nei homolog 1 (NEIL1) glycosylase and apurinic-apyrimidinic endonuclease (APE1) [72,73,74]. Other scenarios are certainly possible and more work is needed to fully understand the sequence of events that leads to expansions.

## 6. Are Expansions and Contractions Reciprocal Events?

Contractions are still seen in FX PM mice with mutations that eliminate expansions completely. Thus, many of the proteins required for expansion are not necessarily required for contraction. Furthermore, expansions only occur when alleles are unmethylated in both mice and humans, whereas methylated alleles can still contract [13], and in humans AGG-interruptions to the purity of the CGG-repeat tract decreases expansions but not contractions [6]. In addition, expansions are also seen in the somatic cells of adult mice and humans, while contractions occur rarely, if at all, in the somatic cells of adult mice [17]. Thus, the preponderance of evidence suggests that expansions and some or all contractions arise by distinct mechanisms.

## 7. What Is Known about Contractions and the Potential for Error-Free Repair?

**Figure 4:**
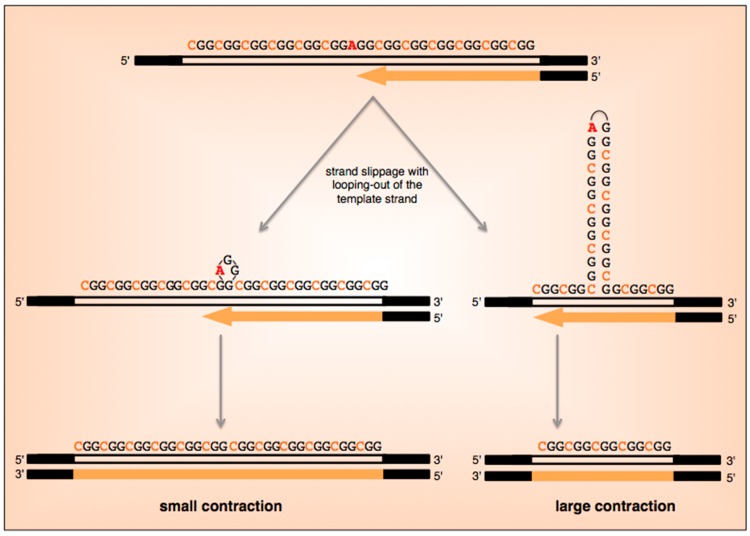
Model for repeat contraction and loss of AGG interruptions. During chromosomal replication or repair synthesis, strand-slippage within the repeat can result in a loop-out forming either on the nascent strand (not illustrated) or the template strand. A loop-out on the slipped nascent strand is thought to result in expansions by causing repriming to occur more 3′ on the template, while a loop-out on the template strand results in repriming more 5′ on the template leading to contractions. In a FX PM mouse model, a bimodal distribution of contraction sizes is seen with some alleles having lost 1–2 repeats and other having lost >7 [48]. Small contractions may result from the looping out of 1–2 repeats while larger contractions arise from the formation of larger loop-outs that may be stabilized by hydrogen bonding. If the template-strand loop-out contains the AGG interruption, then the interruption would be lost.

Very little. Contractions are observed on both maternal and paternal transmission. As with expansions there is direct relationship between repeat number and risk of contraction, with most contracted alleles having been derived from alleles with >36 uninterrupted repeats at the 3′ end of the repeat tract [6]. While the difference in contraction frequency between men and women is not significant below 70 repeats, paternal alleles with 70–90 repeats show a four-fold higher contraction frequency than females [6]. Furthermore, paternally transmitted contractions involved the loss of 10 repeats on average, twice that of females. We have observed contractions occurring at high frequency when ESCs are grown at low plating density [13]. Replication through the repeats is known to be impaired [11,15]. It may be that contractions arise because of the difficulty with progression of DNA polymerases through the repeats under conditions where rapid cell division is required. A similar explanation could account for why FM alleles are not seen in the sperm of FX males. In this case simple strand slippage during replication or repair may account for these contractions as illustrated in Figure 4. A higher frequency of mutational events in males is typically attributed to the larger number of rounds of replication associated with sperm production. This may point to contractions resulting from problems arising during DNA replication. Contractions are frequently associated with the loss of AGG interruptions on maternal but not paternal transmission [6]. Whether this reflects differences in the contraction mechanism in males and females is unclear.

The absence of MutSβ results in more alleles that are the same size as the parental allele than is seen in the absence of MutSα. Furthermore, animals lacking MutSβ show no evidence for the bimodal distribution of contraction sizes seen in wild-type animals having lost all large contractions [41]. However, since large contractions are seen in mice that lack both MutSα and MutSβ, this data suggests that the increase in alleles that are repaired in an error-free way may not reflect a role for MutSβ in generating large contractions. Instead it may indicate that MutSα acts to protect against large contractions, but that for some reason this effect is only apparent when MutSβ is missing. It may be that when MutSβ is present, most of the MutSα is diverted to complexes that result in expansion. The base mismatches that occur in the FX hairpins are reminiscent of the canonical MutSα mismatch repair substrate. It may be that while MutSα contributes to expansions via its interaction with the BER machinery, it protects against contractions via its role in canonical MMR or DNA damage signaling.

Loss of CSB in mice heterozygous for *Msh2*, results in an increased expansion rate suggesting that in addition to contributing to expansions it can under some circumstances also protect against expansion [51]. This apparent paradox can be resolved if CSB is acting in one pathway to promote expansions and in another to prevent them. It may be that MutSα and CSB co-operate in reducing the incidence of some contractions.

## 8. What Can Be Done to Block Expansions or Promote Error-Free Repair or Contractions?

In principle, the repeats can be removed using a genome-editing approach [75]. However, given the issues with delivery of nucleic acids in vivo, whether this would ever be feasible in the postnatal situation is unclear and since there are less risky prenatal approaches to ensuring the birth of healthy children, the use of this approach may be limited.

Small molecules that inhibit expansion or promote contraction or error-free repair are likely to be more easily delivered to appropriate target cells. Inhibiting proteins required for expansion may be one way to block expansion. Histone deacetylase complexes (HDACs) are potentially relevant pharmacological targets since both HDAC3 and HDAC5 promote expansion in a tissue culture model for CTG/CAG-repeat expansions [68]. Furthermore, treatment of a mouse model of HD with an HDAC3-selective inhibitor resulted in both fewer somatic expansions and reduced pathology (Robert Lahue, University of Galway, Galway, Ireland, personal communication, 2016). Whether HDAC inhibitors block expansion of CGG/CCG-repeats would need to be tested. Such inhibitors are already in clinical trials for the treatment of life-threatening diseases. However, since HDACs play an important role in the regulation of a large number of different genes, this approach may lack the necessary specificity. MSH3 has also been suggested as a good target for blocking expansions in other repeat expansion diseases [66,76]. However, the increased risk of disorders such as colorectal cancer for which MutSβ is protective also makes this a risky approach.

Preventing the formation of the structures thought to be the substrates for expansion may provide a more specific, targeted approach to reducing expansions. A pyrrole-imidazole polyamide, FA1, that binds to the FRDA repeats, reduces MSH2 binding in induced pluripotent stem cells (iPSCs) and also reduces expansion [67]. It has been suggested that FA1 acts by eliminating the non-B DNA conformation formed by the repeats [67]. Small molecules that target the FX repeats may be able to act in similar manner.

Finally, since oxidative damage increases expansions in the FX PM mouse [26], antioxidants may have some therapeutic benefit in reducing expansion risk. Since there is evidence to suggest that expression of PM alleles results in mitochondrial abnormalities and increased oxidative stress that may contribute to disease pathology [77,78,79], antioxidants may have an additional benefit in ameliorating some of this pathology. Treatment of an HD mouse with the (*E*)-alkene isostere XJB-5-131, a synthetic antioxidant that targets the mitochondria, was recently shown to both decrease expansions and to ameliorate HD pathology [80]. Similarly, provision of anthocyanin antioxidants in the drinking water of mice showed a modest effect on expansion in the HD mouse cortex [81]. The identification of more effective antioxidants may thus be a useful approach that should be explored.

## 9. Concluding Remarks

In the 25 years that have elapsed since the cause of FXS was first described much has been learnt about the natural history of repeat instability at the *FMR1* locus in human populations, the properties of the repeats in vitro and in vivo, and what factors affect repeat expansion in mouse models. However, many questions remain unanswered. For example, do the factors shown to affect expansion in mice play a similar role in expansion in Fragile X families? If so, how do these factors that normally participate in three different DNA repair pathways, interact to generate expansions? What accounts for the differences in expansion frequencies in different cell types? What factors in addition to X chromosome inactivation (XCI) contribute to the differences in the expansion and contraction frequencies in males and females? Does expansion in dividing cells occur by the same mechanism as it does in non-dividing cells? How is transcription related to expansion? What factors are involved in error-free repair and the generation of contractions? Answers to these questions will help us to better understand the molecular basis of repeat instability at the *FMR1* locus and thus to assess what, if anything, can be done to prevent or reduce expansions in the future.
